# A short report on deep learning synergy for decentralized smart grid cybersecurity

**DOI:** 10.3389/frai.2025.1557960

**Published:** 2025-04-09

**Authors:** Saurav Verma, Ashwini Rao

**Affiliations:** SVKM's NMIMS Mukesh Patel School of Technology Management and Engineering, Mumbai, India

**Keywords:** smart grid, artificial intelligence, machine learning, block chain, deep learning

## 1 Introduction

Smart grid technology is an advanced enhancement of the conventional power grid, characterized by improved communication, control, and computing technologies that optimize energy distribution. A smart grid is an improvement on the existing electric grid system, allowing for more intelligent controlling of electricity from the generation point to the consumer. Consequently, the Industrial Control Systems (ICS) of smart grids have become more exposed to cyber risks due to enhanced network integration and digitalization. The complexity of smart grids, with numerous distributed energy resources (DERs), sensors, and interconnected systems, makes the security problem challenging. The need for secure and resilient decentralized smart grids underscores the importance of new approaches that can enable the detection, prevention, and mitigation of cyber-attacks are desirable. Deep learning and smart grid cybersecurity based on decentralization provide a bright outlook, as they enhance the detection of anomaly cases and potential threats while also increasing the overall resilience of the grid.

## 2 The role of deep learning in cybersecurity

Smart grids represent a major evolution of conventional power grids that employ Information and Communication Technology (ICT) to optimize the delivery of electrical energy. Elements of smart grids include smart meters for consumers, automated distribution networks, and communication networks. Smart grids can be decentralized as it includes multiple DERs such as solar power, windmills, or energy storage systems, which are usually integrated at the edges of the smart grid. Such decentralization introduces additional challenges to the grid's physical structure and also increases the number of vectors through which a cyber threat can penetrate the network (Reuters, 2023). As a result, cybersecurity has emerged as a focal topic to ensure the safe and reliable functioning of smart grids. It has also demonstrated significant success in several contexts, largely due to deep learning's inclusion abilities to extract key features from accessed data (Ghiasi et al., 2023).

A major advantage of deep learning is that models are able to detect the abnormal flow of traffic since they hold knowledge of normal traffic flow patterns (Ortega-Fernandez and Liberati, 2023). Pattern recognition is another important factor, as deep learning networks can identify even complex patterns within a given data. Additionally, deep learning models are scalable, hence making them capable of analyzing large quantities of data produced by, for instance, smart grids (Reda et al., 2022).

## 3 Cybersecurity challenges

Decentralized smart grids are the most vulnerable because of the localized architecture and extensive connections with IoT gadgets. The different systems used in smart grids are not homogenous and have different protocols, which introduce security weaknesses; hence, the weaknesses are present (Thakkar and Lohiya, 2022). The lack of computational capacity in many IoT devices due to resource constraints precludes such approaches, and traditional security techniques cannot be implemented (Xia et al., 2022).

Despite the potential of deep learning in smart grid cybersecurity, there are a number of issues to address. Concerning a few key points, it is important to mention data confidentiality, as the training data can be considered sensitive. Another issue is the interpretability problem since, unlike traditional machine learning techniques, deep learning models are considered “black box” (Verma and Prakash, 2023). In addition, a limitation of deep learning is that it demands massive computation, which may not be readily feasible in low-power devices of smart grids (Vosughi et al., 2022). There is also a significant issue related to integration with legacy systems, as such systems might be incompatible with deep learning solutions (Esenogho et al., 2022).

## 4 Future directions

**Figure 1:**
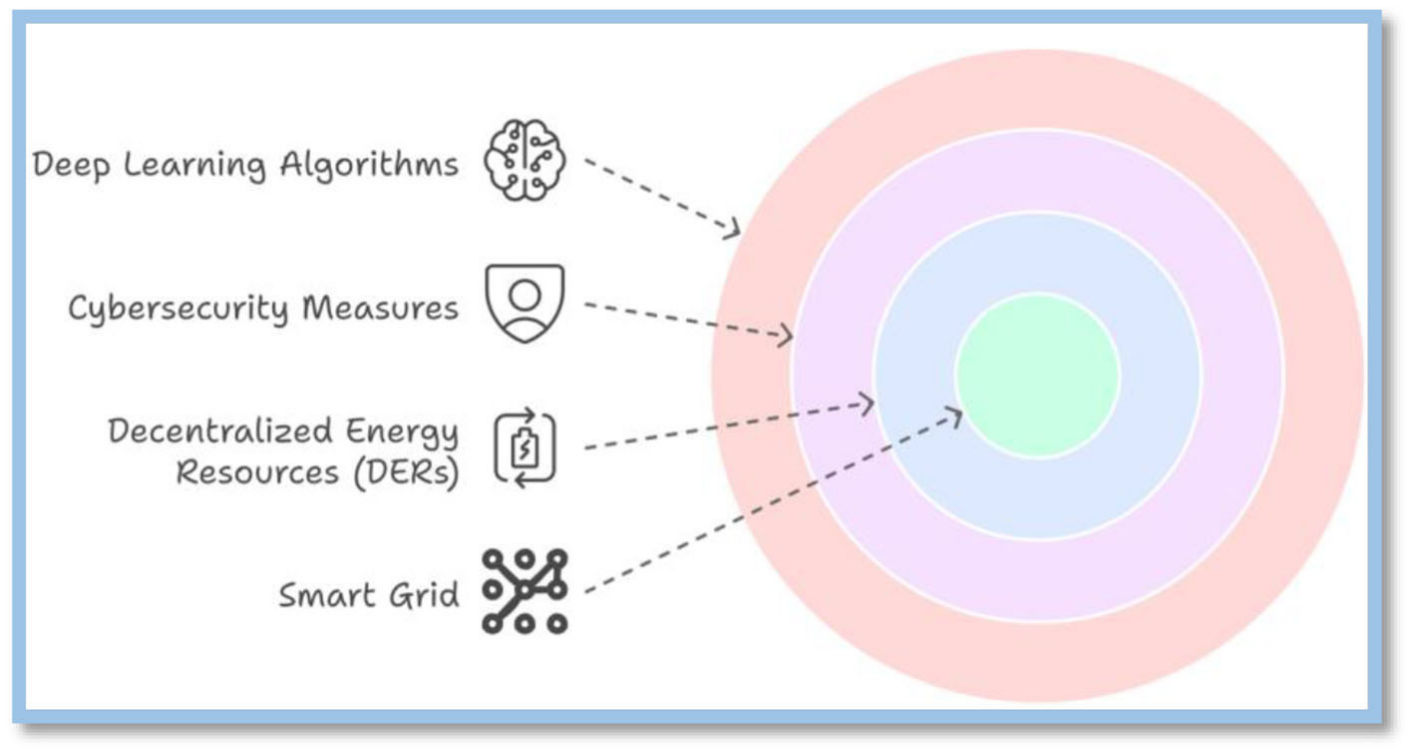
Smart grid and cybersecurity integration.

Future research directions encompass the development of smart grids that are decentralized and experience a higher level of risk related to cybersecurity because of the deployment of several IoT devices. Table 1 summarizes robust deep learning approaches for smart grid cybersecurity, highlighting their advantages, challenges, and future directions. While these methods show high accuracy in detecting cyber threats (ranging from 92% to 99.5%), they face issues such as high computational demands, vulnerability to adversarial attacks, and scalability concerns. Future research should focus on improving real-time integration, enhancing model interpretability, and developing more robust AI-driven cybersecurity frameworks as shown in Figure 1.

**Table 1:** Analysis of the robust methods of deep learning for decentralized smart grid cybersecurity.

**Reference**	**Method**	**Advantages**	**Challenges**	**Evaluation metrics**	**Future directions**
Lahon et al. (2024)	Transformer-based Deep Neural Networks (DNN) for Smart Grid Stability Analysis	Better handling of complex datasets; Improved sequential pattern recognition	High computational demand; Interpretability issues	Accuracy: 99.5%, Precision: 98%, Recall: 97%, F1-Score: 97.5%, Latency: Medium	Integration with real-time monitoring systems and validation through large-scale smart grid simulations
Ruan et al. (2023)	Graph Neural Networks (GNN) + Adversarial Learning for Smart Grid Cybersecurity	Improved threat detection in complex networked systems	Vulnerability of DL models to adversarial attacks; High training complexity	Accuracy: 97%, FPR: 4.2%, Precision: 96%, Recall: 95%, F1-Score: 95.5%	Development of adversarially robust GNN-based cybersecurity frameworks
Nemade et al. (2024)	Hybrid AI-based Cyber Defense (Deep Learning + Expert Systems)	Better anomaly detection; Enhanced SCADA system protection	Scalability and interoperability issues; High dependency on rule-based logic	Accuracy: 95%, F1-Score: 94.2%, Precision: 94%, Recall: 93.5%, Latency: Low	Strengthening AI-driven threat detection with continuous learning from real-world cybersecurity incidents
Manias et al. (2024)	Federated Learning for Smart Grid Security	Enables decentralized learning without sharing raw data; Privacy-preserving	Communication overhead; Vulnerability to poisoning attacks	Accuracy: 92%, FPR: 5.1%, Precision: 90%, Recall: 91%, F1-Score: 90.5%, Latency: Medium	Development of secure federated learning models with blockchain-based verification
Boopathy et al. (2024)	AI and Blockchain-based Intrusion Detection System	Improved data integrity and security; Enhanced resilience against tampering	High resource consumption; Latency issues in real-time operations	Accuracy: 92%, F1-Score: 92%, Precision: 93%, Recall: 91%, Latency: High	Optimization for real-time efficiency and scalable, lightweight blockchain security solutions
Kotsiopoulos et al. (2024)	Adversarially Trained Deep Learning for Smart Grid Cybersecurity	Enhanced robustness against evasion attacks; Handles high-dimensional data	High computational requirements; Requires extensive adversarial training	Accuracy: 94%, FPR: 4.8%, Precision: 92%, Recall: 91.5%, F1-Score: 91.8%, Latency: Medium	Improved model interpretability and deployment strategies for edge computing with adversarial robustness
Mohammadpourfard et al. (2021)	Hybrid CNN-RNN for Attack Detection and Localization	No prior statistical assumptions required; Effective anomaly localization	Computational cost; Dependence on real-world datasets	Accuracy: 98%, Precision: 97.5%, Recall: 97%, F1-Score: 97.2%, Latency: Medium	Further enhancement using hybrid models combining CNN, RNN, and reinforcement learning
Ruan et al. (2023)	Self-Supervised Learning (SSL) for Smart Grid Cybersecurity	Reduces dependency on labeled data; Improved generalization to unknown threats	High complexity; Requires large-scale pre-training	Accuracy: 96%, FPR: 3.9%, Precision: 95%, Recall: 94.5%, F1-Score: 94.7%, Latency: Low	Development of SSL-driven AI frameworks for real-time, adaptive cybersecurity defense

The most suitable solution for smart grid cybersecurity protection combines federated learning, blockchain, and adversarial deep learning. With FL, grid nodes can participate in decentralized training processes without exchanging the actual data, thereby protecting their information security and privacy. The combination of blockchain technology and adversarial deep learning training creates an unalterable security framework that secures communication while building resistance against complex cyber threats. The implementation of this combined method becomes necessary because smart grids function through decentralized systems that connect numerous vulnerable IoT-enabled energy production networks to cyber security threats. Maximum security models become ineffective because they suffer from dimensional problems, privacy weaknesses, and developing electronic strike threats. Through an integration of FL and blockchain technology, organizations achieve real-time threat detection with adaptive capabilities and lower IT overhead costs. Industrial security in the energy sector needs sophisticated AI-enabled solutions that must scale effectively to defend against infrastructure attacks and disruptions to the power supply. By utilizing this model, organizations maintain autonomous cybersecurity operations that produce efficient proactive threat protection suitable for advanced smart grid systems against developing cyber-attacks.

## 5 Conclusion

Deep learning in decentralized smart grid cybersecurity is a revolutionary approach of handling vast and dynamic risks. Based on the real-time data processing capabilities and the pattern recognition ability of deep learning models, it is possible to improve anomaly detection density, threat prediction, and system robustness. However, challenges exist in using deep learning in this area, including the need for significant computational power, data security concerns, and difficulties related to the initiation and incorporation of integration of such complex technologies into existing systems. To overcome these challenges, new approaches must be developed more efficiently, with a focus on building effective privacy preservation mechanisms and ensuring seamless integration with other existing systems. Future work should focus on introducing sophisticated and flexible deep learning frameworks for smart grids that can function in a given distributed environment. This will enable the industry to progress and advance toward building a smarter grid that can address novel cyber threats and protect and enhance the reliability of energy distribution systems.
